# Portable Dynamic Laser Speckle Imaging for Rapid Antimicrobial Susceptibility Testing

**DOI:** 10.1149/2754-2726/ae1e10

**Published:** 2025-12-03

**Authors:** Jinkai Yang, Keren Zhou, Landon Hernandez, Chen Zhou, Olena Voloshchuk, Jasna Kovac, Aida Ebrahimi, Zhiwen Liu

**Affiliations:** 1 School of Electrical Engineering and Computer Science, The Pennsylvania State University, University Park, Pennsylvania 16802, United States of America; 2 Materials Research Institute, The Pennsylvania State University, University Park, Pennsylvania 16802, United States of America; 3 Department of Food Science, The Pennsylvania State University, University Park, Pennsylvania 16802, United States of America; 4 Biomedical Engineering, The Pennsylvania State University, University Park, Pennsylvania 16802, United States of America

## Abstract

Antimicrobial resistance remains a pressing global health threat. Conventional antimicrobial susceptibility testing (AST) methods are limited by long incubation times and centralized laboratory requirements, hindering timely decision-making in resource-limited settings. This study introduces a 3D printed, portable dynamic laser speckle imaging (pDLSI) device for rapid AST. This device has a 10 × 3 × 3 cm^3^ footprint, equipped with a low-cost laser diode, a lens assembly, and a cuvette sample holder. Speckle fluctuations induced by bacterial activity are captured on a cellphone camera, visualized using spatiotemporal decorrelation maps, and analyzed using machine learning to determine minimum inhibitory concentrations within 2–3 h. To demonstrate the performance, we studied two representative Gram-positive and Gram-negative bacterial strains (*Enterococcus faecalis* and *Escherichia coli*), along with two antibiotics (ampicillin and gentamicin). The simple construction, portability, and intuitive operation of the pDLSI system can potentially facilitate initial diagnostic decisions at the point of care.

Antimicrobial resistance (AMR) remains a critical threat to global public health,^1–3^ with projections suggesting AMR could result in 10 million deaths annually by 2050.^4^ The highest mortality rates are projected in Asia, followed by Africa, where large populations in resource-limited settings make it challenging to prevent the spread of resistance.^5,6^ Antimicrobial susceptibility testing (AST) plays a critical role by enabling clinicians to select effective antibiotics and reduce inappropriate prescriptions that drive resistance.^7^ AST techniques determine the minimum inhibitory concentration (MIC), or the lowest concentration of an antibiotic required to inhibit bacterial growth.^8^ However, conventional AST techniques, such as broth microdilution, disk diffusion, and gradient diffusion, are inherently time-consuming and labor-intensive.^9^

Portability in healthcare delivery is crucial, particularly in settings with limited access to medical laboratory resources.^10^ In regions with large or underserved populations, where fixed laboratory infrastructures are insufficient to meet demand, portable diagnostic tools allow for flexibility and rapid response.^11–14^ Several studies have proposed portable diagnostic platforms for rapid AST, including droplet-based impedance sensors,^15,16^ electrochemical methods,^17,18^ and platforms based on printed electrodes.^19,20^ Optical sensing techniques, with their high sensitivity, lightweight, cost-effectiveness, and compact designs, are particularly well-suited for miniaturization into portable diagnostic devices.^21–25^ For example, cellphone-based detection systems have been explored for portable AST, such as microplate readers that rely on turbidity detection,^26^ which, while promising, require overnight incubation. On the other hand, cell counting using imaging methods depends heavily on the performance of microfluidic systems, which require precise optimization of channel geometry, surface properties, and flow conditions to accommodate different bacterial species and experimental environments.^27^

Here, we present a portable dynamic laser speckle imaging (pDLSI) system that extracts bacterial viability information through motion analysis. This portable system builds upon our previous work on the dynamic laser speckle imaging (DLSI) platform,^28^ which was developed on an optical table using research-grade devices and instrument. The pDLSI provides a compact, portable design at a significantly lower cost: the overall cost is approximately 18 times lower (*see* Supplementary Information—System Cost Comparison). The pDLSI system consists of a laser diode, a three-lens assembly, and a cuvette-based sample holder, all enclosed within a 3D-printed housing. Dynamic speckle patterns are projected onto a paper screen and captured using an iPhone camera. Compared with our previous DLSI system, which was implemented on an optical table using research-grade instruments and precision-aligned components, the pDLSI system achieves comparable sensitivity while significantly reducing the overall system size, cost, and operational complexity. In addition, we introduced a method for assessing speckle spatiotemporal pattern by computing the temporal decorrelation, providing an intuitive visualization of bacterial susceptibility. This spatiotemporal mapping provides a quick tool for preliminary data interpretation. Our proof-of-concept portable system has been tested with ~5 × 10^5^ CFU ml^−1^ of Gram-negative (*E. coli*) and Gram-positive (*E. faecalis*) species, demonstrating its effectiveness in rapid prediction of antibiotic susceptibility (*see* Supplementary Information—PCA Visualization). This study establishes a pathway toward practical, field-deployable antimicrobial susceptibility testing and broadens the applicability of dynamic speckle imaging for portable biosensing platforms.

## Results and Discussion

**Figure 1.**
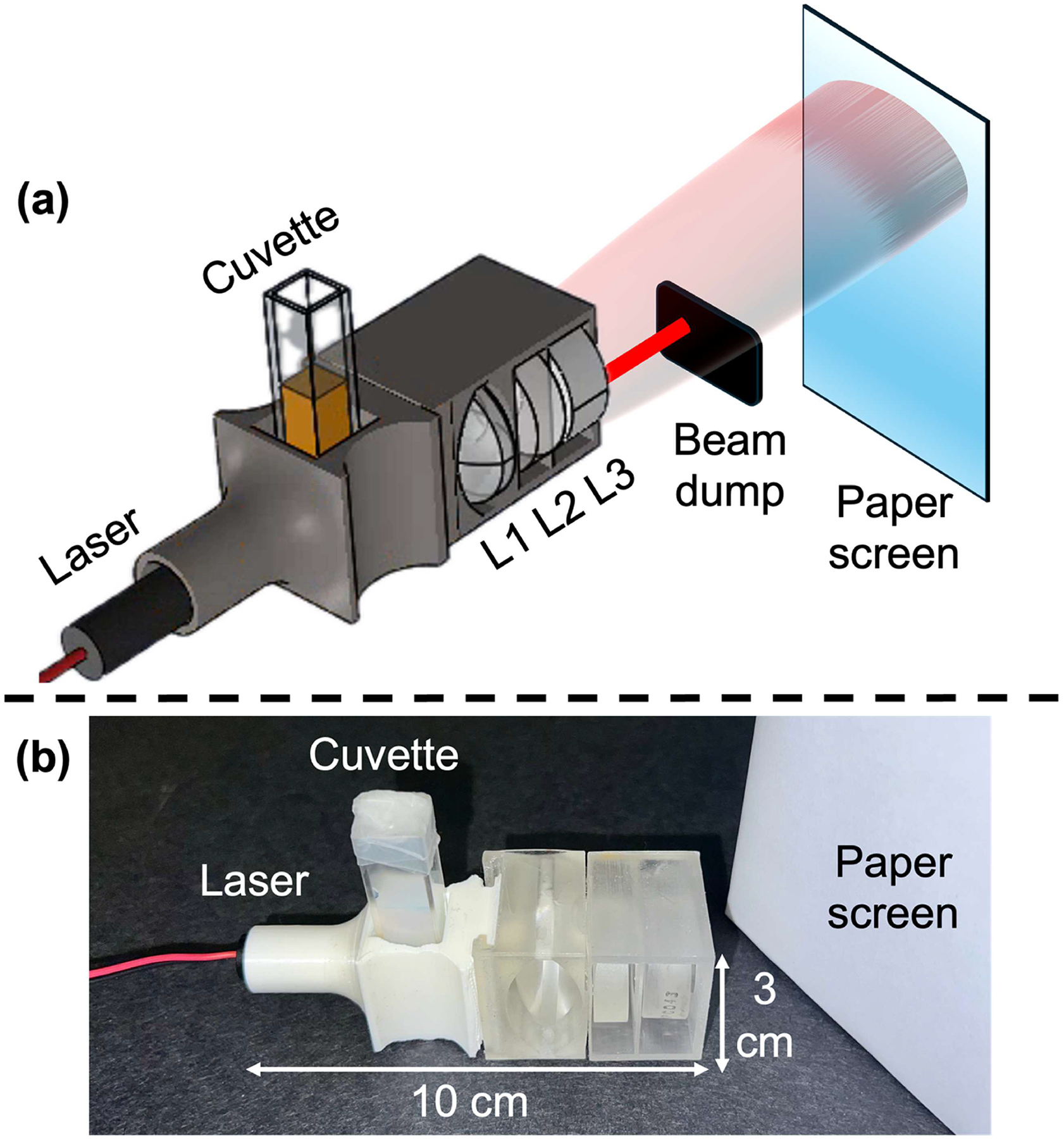
Schematic of the portable Dynamic Laser Speckle Imaging system. (a) A laser diode emits a beam that passes through a cuvette containing the sample suspension. Scattered light from the sample is collected by a lens system (L_1_, L_2_, L_3_) and projected onto a paper screen, where dynamic speckle patterns form. The residual laser beam is blocked by a beam dump to enhance contrast. These speckle patterns are captured by a cellphone camera and analyzed to monitor bacterial motion in response to antibiotic exposure. (b) A photo of the pDLSI device. After 3D printing and assembly, the physical dimensions of the prototype were measured to be approximately 10 cm × 3 cm × 3 cm.

Figure 1 shows a schematic of the pDLSI system and a photo of the device. The system uses a 650 nm laser diode with a measured output power of 3.3 mW. The laser beam is directed onto a cuvette containing a sample suspension, where the laser light is scattered by the sample. The scattered light is collected by a lens system (L_1_, L_2_, L_3_) and directed toward a white printer-paper screen, forming speckle patterns. The use of the paper viewing screen provides a low-cost approach to mitigate vignetting and increase the field of view. The residual incident laser beam is blocked by a beam dump to prevent oversaturation and preserve contrast in the speckle image. A cellphone camera records the evolution of these speckle patterns from the paper screen. For bacterial suspension, these dynamic speckle patterns provide crucial information about bacterial motion, which is linked to the bacteria’s response to antibiotics. If bacteria are actively moving (non-inhibited), the speckle pattern changes rapidly. In contrast, if bacterial motion is reduced due to antibiotic inhibition, these fluctuations become less pronounced. The temporal characteristics of the speckle patterns are analyzed to assess the bacterial susceptibility to antibiotics.

The pDLSI device includes a laser diode mount, a sample cuvette holder, and a lens combination mount into a 3D-printed lightweight structure. Its overall size, approximately 10 cm in length and 3 cm in both width and height, supports easy handling and portability. Our design uses off-the-shelf optical components and standard cuvettes, without the need for costly and complex laboratory equipment. The ability to directly probe cuvette samples allows bacterial samples in their native liquid state, preventing desiccation and preserving sample viability, which are critical for reliable AST outcomes. The simplicity of this device also reduces operational complexity, requiring minimal optical alignment and technical expertise.

**Figure 2.**
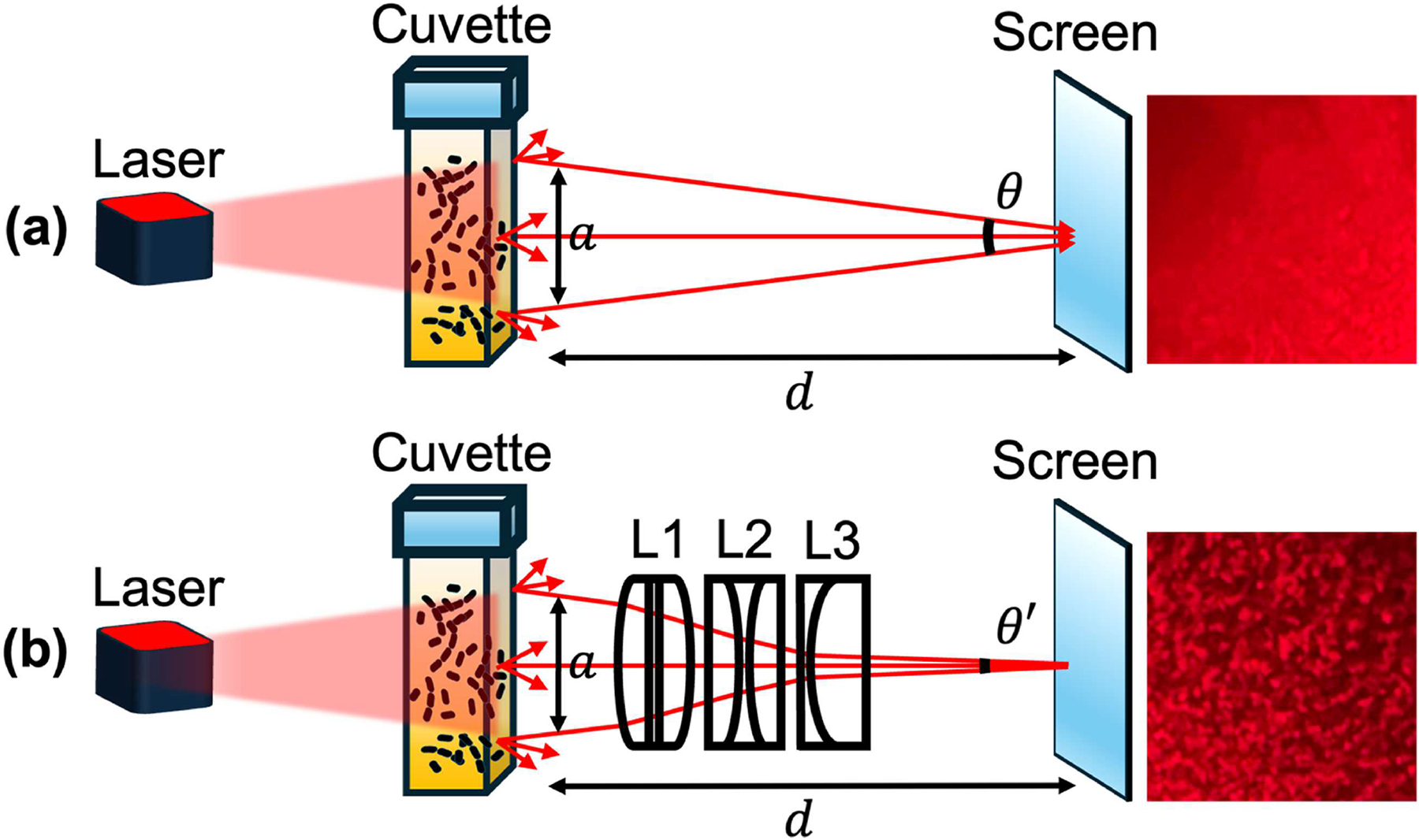
Schematic and experimental validation of speckle grain enlargement by the lens system. (a) Without the lens system, the scattering medium (illuminated by the laser beam) has an angular size *θ*. Since speckle grain size is inversely proportional to the angular size, a larger *θ* produces finer speckle grains, as shown in the top panel. (b) Inserting the lens assembly (L_1_−L_3_) reduces the apparent angular size to *θ*′, which is smaller than *θ*, increasing the speckle grain size. This effect is confirmed experimentally in the bottom panel, where enlarged speckle grains are observed.

Figure 2 illustrates the schematic and experimental validation of speckle grain enlargement due to the lens system. As shown in Fig. 2a, without the lens system, the speckle patterns formed directly on a white printer-paper screen exhibit small grain sizes. Because individual speckles cannot be resolved, the resulting image shows poor contrast and appears as a uniform, low-contrast smear, not suitable for quantitative analysis. Note that the average laser speckle grain size $\approx \frac{\lambda *d}{a}$, where *λ* is the illumination wavelength, *d* is the propagation distance from the scattering medium to the detection plane, and *a* is the effective size of the scatterer.^29–31^ Thus, larger speckle grains can be obtained either by increasing *d* or by decreasing *a*. The speckle size relationship can also be rewritten as $\approx \frac{\lambda *d}{a}=\frac{\lambda }{\theta }$, where *θ* represents the apparent angular size of the scatterer. To maintain a compact device footprint, *θ* can be reduced to a smaller angle, thereby enlarging the speckle grain size. A raytracing simulation (Fig. 2b, left) demonstrates that the lens system (L_1_–L_3_) effectively reduces the apparent angular size to a smaller value *θ*′. Experimental validation (Fig. 2b, right) confirms that this lens system increases the speckle grain size compared to the lens-free configuration, in agreement with simulation predictions. Additionally, to minimize the risk of sample damage due to direct laser heating, we use a low-power laser diode as the illumination source. At the same time, it is essential to ensure that the collected light intensity is sufficient for reliable speckle pattern acquisition. Our optical design enhances the system’s ability to resolve speckle dynamics, enabling reliable monitoring of bacterial motion and antibiotic susceptibility under safe, low-intensity illumination.

**Figure 3.**
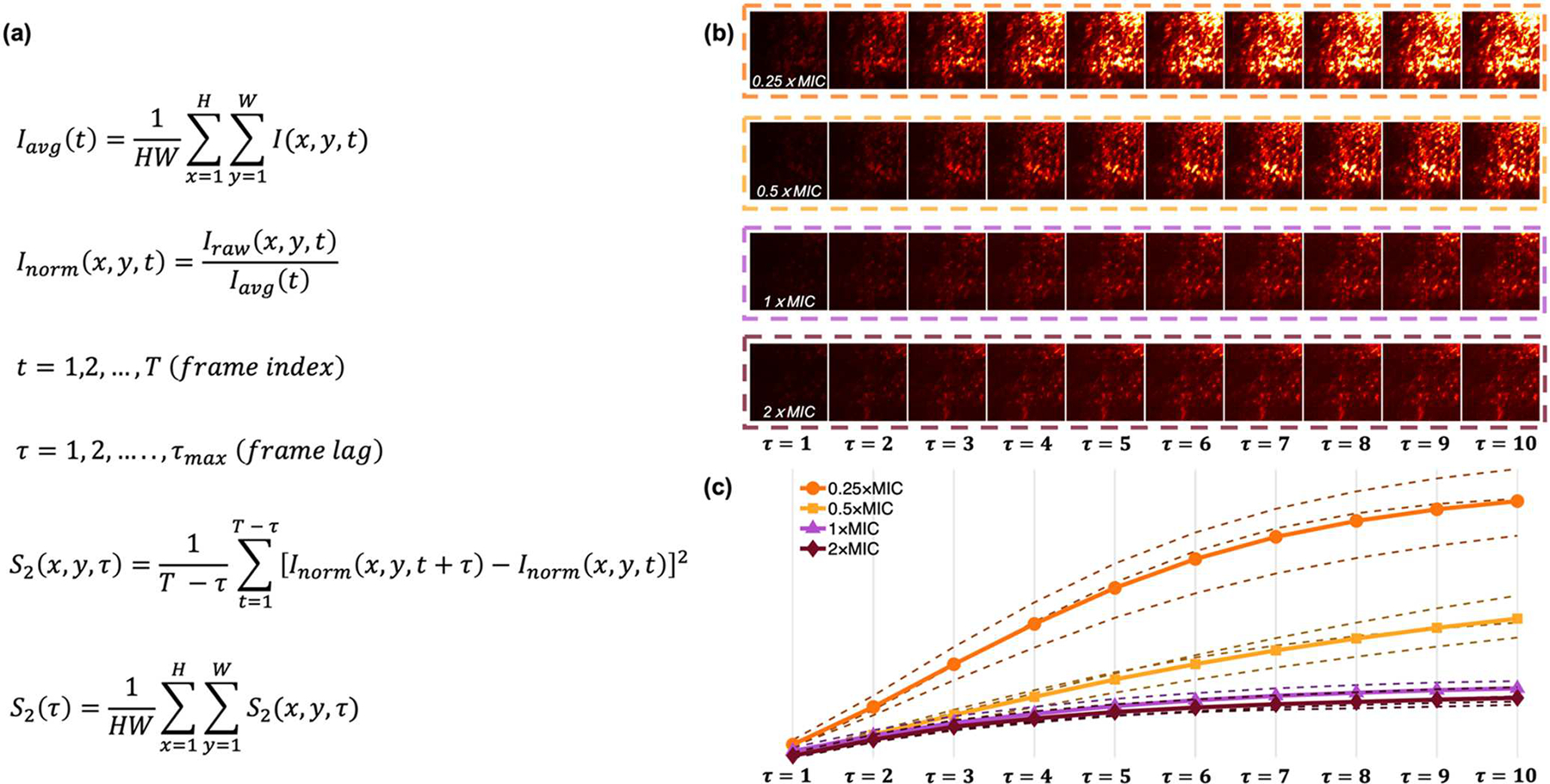
Visualization of bacterial-motion induced speckle dynamics. (a) I(x,y,t) represents the pixel intensity at position (x,y) and frame index t. H and W denote the frame height and width, respectively, and T is the total number of frames. The normalized intensity I_norm_(x,y,t) is obtained by dividing each frame by its spatial average I_avg_(t). S_2_(x,y,τ) quantifies the intensity fluctuations between frames separated by a frame lag τ. (b) Representative S_2_(x,y,τ) obtained from bacterial samples treated with different antibiotic concentrations (0.25× MIC to 2× MIC) across increasing frame lags (τ = 1–10 frames). Higher S_2_ values correspond to stronger temporal intensity variations, indicating greater bacterial mobility. (c) Spatially averaged curves S_2_ derived from each antibiotic concentration, illustrating progressively slower temporal decorrelation as the antibiotic concentration increases.

We tested the performance of pDLSI with *E. coli* and *E. faecalis* samples prepared at an initial concentration of ~5 × 10^5^ CFU ml^−1^, a standard concentration used in the broth microdilution protocol by Clinical and Laboratory Standards Institute (CLSI) for determining susceptibility or resistance based on the breakpoint concentrations.^32^ Four antibiotic concentrations—0.25 × MIC, 0.5 × MIC, 1 × MIC, and 2 × MIC—were used in our studies, each verified through broth microdilution (details are discussed in Materials and Methods). To perform an initial assessment of the bacterial response to applied antibiotics, in Fig. 3a we present a method to visualize distinct speckle dynamic behaviors associated with different bacterial inhibition statuses. For each video, a spatially averaged intensity is first computed for every frame. Each frame is then normalized by its corresponding spatial average to eliminate potential power fluctuations caused by illumination instability or other unwanted effects. Next, the temporal evolution is quantified by computing the difference between image pairs separated by a frame lag τ, where τ ranges from 1 to 10 frames. For instance, when τ = 2, we compare frame 1 with frame 3, frame 2 with frame 4, and so on, up to frame t and frame t + τ. Averaging the squared differences across all pairs with the same τ produces a map S_2_(x,y,τ), which represents the spatial distribution of temporal intensity fluctuations at that frame lag. Figure 3b presents the resulting maps for *E. coli* samples exposed to different concentrations of gentamicin after three hours of incubation. At τ = 1, the four concentration groups exhibit similar patterns, indicating limited temporal decorrelation within short time intervals. As τ increases, the maps of sub-MIC treated samples show stronger intensity variations and richer spatial structures, reflecting rapid speckle decorrelation associated with active bacterial motion. In contrast, samples treated at or above the MIC exhibit much weaker intensity fluctuations or slower decorrelation, consistent with inhibited bacterial activity. Figure 3c displays the spatially averaged curves S_2_(τ) for each concentration. These curves reveal a clear concentration-dependent trend, where higher antibiotic concentrations lead to slower temporal decorrelation, confirming the suppression of bacterial motion under effective antimicrobial treatment. This spatiotemporal mapping serves as an intuitive visualization tool for preliminary data interpretation.

As clearly shown from the observed speckle dynamics, inhibited and non-inhibited bacterial samples can be differentiated based on their distinct speckle signatures. Building on our previous work,^33^ we convert the temporal speckle sequences into Fourier-domain dataset to help extract frequency response features suitable for machine learning. An artificial neural network is then trained to learn the relationship between speckle evolution characteristics and bacterial inhibition state. Once trained, the model can determine the minimum inhibitory concentration by recognizing differences in speckle evolution patterns associated with inhibition.

**Figure 4.**
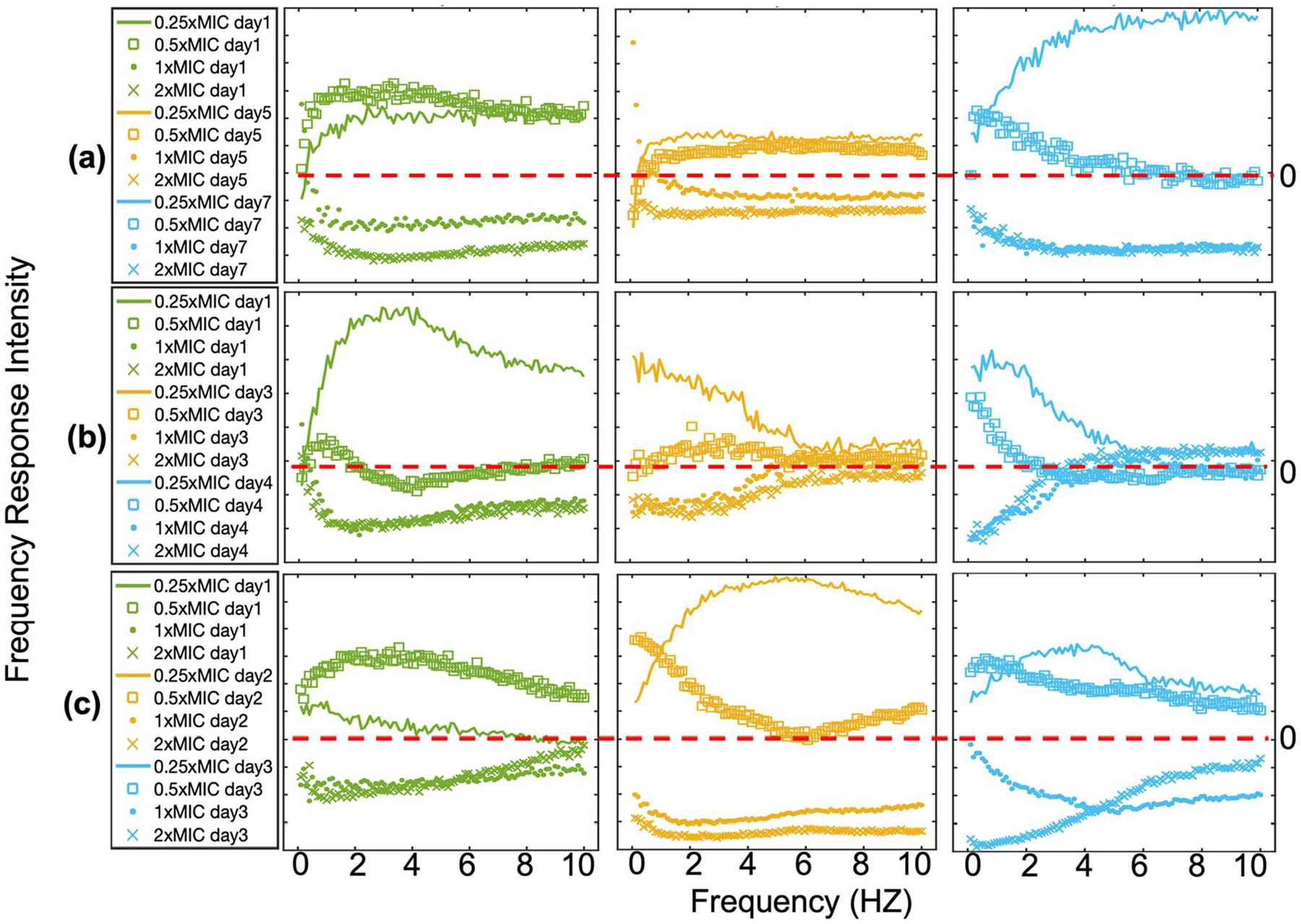
Z-score normalized average frequency response plots for bacterial cultures treated with various antibiotic concentrations, showing changes in frequency response over time. (a) *E. coli* exposed to ampicillin at an initial concentration of ~5 × 10^5^ CFU ml^−1^ for 2 h, (b) *E. coli* exposed to gentamicin at an initial concentration of ~5 × 10^5^ CFU ml^−1^ for 3 h, and (c) *E. faecalis* exposed to ampicillin at an initial concentration of ~5 × 10^5^ CFU ml^−1^ for 3 h. The frequency responses are plotted for each antibiotic concentration: 0.25×MIC (solid line), 0.5×MIC (square line), 1×MIC (solid dot), and 2×MIC (cross sign). Each plot shows data from independent experiments conducted on separate days, as represented by different colors. These results show the reproducibility of bacterial response to antibiotic treatment across multiple experimental trials and demonstrate how average frequency response intensity varies with antibiotic concentration and bacterial susceptibility.

To evaluate the reproducibility of the pDLSI, we conducted independent experiments in different days for each bacteria-antibiotic pairing. For each antibiotic concentration, we also performed triplicate measurements. Experiments involving *E. coli* with ampicillin were conducted on days 1, 5, and 7, while experiments for *E. coli* with gentamicin were conducted on days 1, 3, and 4. Similarly, experiments for *E. faecalis* with ampicillin were conducted on days 1, 2, and 3. Figure 4 presents the frequency response profiles observed for each bacteria-antibiotic concentration. To ensure comparability, the same normalization process, similar to Z-score, was applied to all datasets (data normalization techniques are discussed in Materials and Methods). Regardless of the test day or bacteria-antibiotic pairing, suspensions treated with sub-MIC levels (0.25× and 0.5×) consistently showed positive intensity after normalization, while those at MIC or higher (1× and 2×) had negative readings. This consistency demonstrates the portable system’s capability to differentiate inhibited samples from non-inhibited ones.

**Figure 5.**
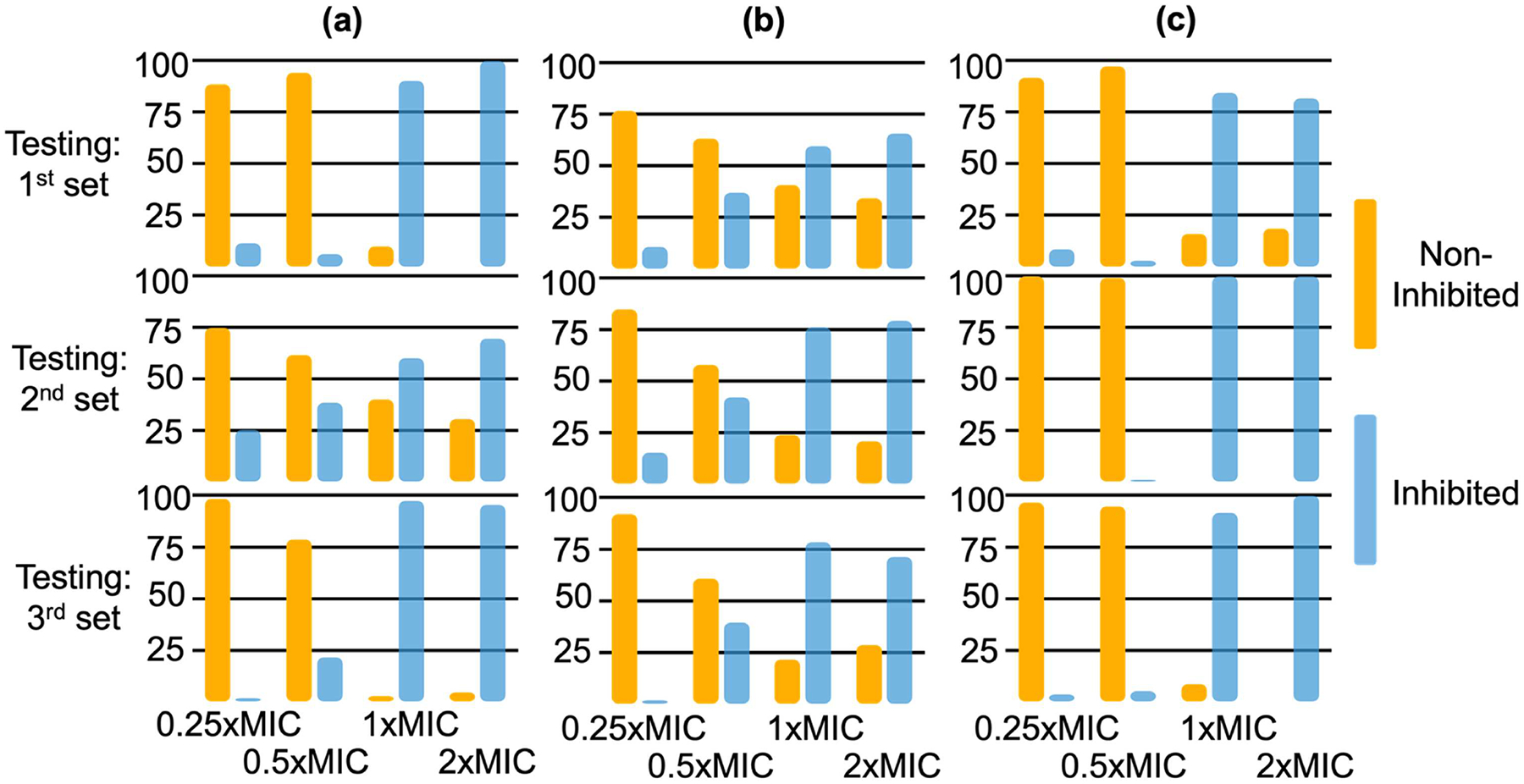
Machine learning prediction results for bacterial cultures treated with varying concentrations of antibiotics. (a) Ampicillin treatment of *E. coli* with an initial concentration of ~5 × 10^5^ CFU ml^−1^ for 2 h, (b) gentamicin treatment of *E. coli* with an initial concentration of ~5 × 10^5^ CFU ml^−1^ for 3 h, and (c) ampicillin treatment of *E. faecalis* with an initial concentration of ~5 × 10^5^ CFU ml^−1^ for 3 h. The results are shown for three independent testing sets. The first row presents prediction results from a model trained on data from the second and third independent experiments, tested on unseen data from the first experiment. The second row shows prediction results from a model trained on data from the first and third experiments, tested on unseen data from the second experiment. The third row demonstrates prediction results from a model trained on data from the first and second experiments, tested on unseen data from the third experiment. The non-inhibited (orange) and inhibited (blue) categories are clearly distinguished across antibiotic concentrations and experimental sets, demonstrating the model’s ability to generalize across different conditions.

Finally, we used ANN to quantify bacterial susceptibility and determine the MIC values. Each bacteria-antibiotic pairing was evaluated through three independent experiments conducted on different days. For each case, an ANN model was trained on data from two of the three days and tested on the remaining day, with all possible combinations of training and testing sets considered (*see* Supplementary Information—Prediction Results). This testing strategy demonstrates the model’s adaptability to experimental variations with different datasets for training and testing. All data were labeled based on the broth microdilution method, where MIC represents the lowest antibiotic concentration that prevents bacterial growth. MIC determination involves exposing bacteria to a range of antibiotic concentrations and monitoring growth after an incubation period of 16–20 h. After MIC was identified through broth microdilution, AST was performed using our pDLSI system on *E. coli* and *E. faecalis* at concentrations of ~5 × 10^5^ CFU ml^−1^. Figure 5 illustrates the model’s predictions for bacterial cultures across the different antibiotic concentrations and experimental conditions. The first row displays predictions from models trained on data from days two and three and tested on data from day one. The second row shows models trained on data from days one and three, tested on data from day two. The third row represents models trained on data from days one and two, tested on data from day three. The clear separation between inhibited (blue) and non-inhibited (orange) categories across various concentrations and conditions demonstrates the robustness of our approach in accurately classifying bacterial susceptibility across experimental variations.

## Conclusions

The increasing global threat of AMR necessitates the development of diagnostic tools that can deliver timely, accurate results in a wide range of healthcare settings. Our pDLSI system addresses this challenge by providing rapid AST without reliance on costly and complex laboratory equipment. Tested with representative bacterial strains and antibiotics, this system shows both robustness and versatility. Its compact design, combined with spatiotemporal decorrelation visualization and machine learning-enhanced analysis, positions it as a portable tool for resource-limited environments, where conventional AST methods are less practical.

While the present work prioritizes portability and low cost by employing a simple optical detection system, this design inherently trades off some sensitivity. Enhancing detection performance through portable implementations of holographic laser speckle imaging will be a future direction for development. Future work will also aim to broaden the scope to include a wider range of antibiotic classes, as well as to validate the system using clinical samples. Finally, although the device is portable, its compatibility with clinical decision-making workflows requires further investigation. If future work can overcome current limitations in sensitivity, antibiotic coverage, and clinical validation, the system has the potential to improve patient outcomes, support antimicrobial stewardship, and help mitigate the spread of drug-resistant infections.

## Materials and Methods

### Bacteria cultures and antimicrobial susceptibility testing assays.—

The bacterial culture and AST sample preparation procedures used in this study follow protocols consistent with our previous work.^33^ Briefly, *Escherichia coli* K-12 (ATCC 10798) and *Enterococcus faecalis* (ATCC 47077), were selected as representative Gram-negative and Gram-positive bacteria. Microbial cultures were cryopreserved at −80 °C. Strains were resuscitated by streaking onto Mueller Hinton agar plates, followed by incubation at 35 °C. The cultures were then diluted in Mueller Hinton II broth to achieve the final concentration of ~5 × 10^5^ CFU ml^−1^. For susceptibility testing, ampicillin and gentamicin were prepared following CLSI guidelines, and the MICs were determined using the broth microdilution method. Each assay was performed in at least three independent biological replicates, with each including two technical replicates. Cultures were incubated at 35 °C, and 2.5 ml samples were taken from each culture every hour for portable system imaging. After each imaging session, the samples were discarded. More detailed descriptions of the culture conditions, MIC assay protocol, and sample preparation process can be found in our previous publication.

### Portable optical system.—

A compact, portable optical system was designed for rapid antimicrobial susceptibility testing. A laser diode (DigiKey 1528–1391-ND; length: 3 cm, body diameter: 1 cm) was selected as the illumination source to minimize the device footprint. The cuvette containing the bacterial suspension was positioned directly after the laser diode. The lens assembly comprises one convex lens (Newport KBX043) placed approximately 3 cm from the cuvette center, followed sequentially by two concave lenses (Thorlabs LD2297-A and Newport KPC043), forming a three-lens system (L_1_, L_2_, L_3_) to enhance the speckle grain size. Inside the cuvette, the incident laser beam undergoes scattering within the sample suspension. The scattered light is collected by the lens assembly, projected onto a white printer-paper screen placed 20 cm away from the lens system, and captured by a smartphone camera (iPhone 13 Pro) at 24 frames per second. A greater distance between the paper and the lens system results in larger speckle grains but reduces the captured intensity due to power spreading over a larger area. The low-cost paper viewing screen mitigates vignetting to achieve a large field of view. Alternatively, a field lens could also be used; such a method would, however, increase complexity and cost. Although scattering by the rough paper surface introduces additional phase modulation, this contribution remains largely static over the recording time. Consequently, in our application it does not significantly interfere with the dynamic speckle analysis, which only tracks temporal variations in speckle patterns resulting from bacterial motion. Both the paper screen and the smartphone camera need to remain stationary during recording to prevent unwanted motion artifacts that could mimic dynamic speckle fluctuations. Depending on the laser power, low ambient lighting conditions may also be required to ensure sufficient speckle contrast. To prevent oversaturation of the camera and preserve speckle contrast, the residual incident laser beam was blocked by a beam dump placed along the optical axis. This beam dump can be either commercially purchased or homemade. In our experiment, a metal razor blade was used. While the current system demonstrates functionality and portability, future refinements will target improvements in lens configuration, further device miniaturization, and enhancement of overall system sensitivity.

### Data collection, pre-processing, and machine learning model development.—

The data collection protocol and analysis methods follow closely our previous work.^33^ An iPhone 13 Pro captured a sequence of 240 frames. Temporal data at each pixel was converted into the frequency domain using Fourier transform, retaining only positive frequencies in the 0.1 Hz to 10 Hz range, where the most pronounced differences between inhibited and non-inhibited bacterial cultures appeared. Our analysis relied solely on the magnitude of the frequency components, while the spectral phase information was not included. Each Fourier spectrum was normalized to its DC (0 Hz) component. Additionally, a normalization approach similar to z-score was applied. This calibration improved consistency, allowing for meaningful comparison of data collected at different experiments across different days (*see* Supplementary Information—Normalization Methods). The dataset used to train the ANN model was generated from two independent experiments conducted on different days. For each experiment, three technical replicates were recorded for each of the four antibiotic concentrations, with each video containing 500 × 500 pixels of frequency response data. This resulted in approximately 6,000,000 data points, which were compiled and processed for training the ANN model using MATLAB’s Classification Learner. The ANN model architecture has an input layer with 100 neurons for the frequency components (0.1 to 10 Hz), a hidden layer of 100 neurons with ReLU activation, and a binary output layer to classify “inhibited” vs “non-inhibited” samples. Data from two of the three experimental days were used for training, with an 80/20 split, where 80% of the data was allocated for model training and 20% for holdout validation. For testing, the trained model was evaluated on the third day’s dataset, consisting of 3,000,000 unseen data points. Each pixel was classified individually, and an overall classification for each sample was determined using a majority voting approach. If more than 50% of the pixels were classified as either “inhibited” or “non-inhibited,” the corresponding label was assigned to the entire sample.

## Supplementary Material

SI

Supplementary material for this article is available online
