# Supplementary material for: Portable Dynamic Laser Speckle Imaging for Rapid Antimicrobial Susceptibility Testing
Source: ECS Sens Plus. Author manuscript; Available in PMC 2026 Jan 29. (PMC12851625; doi:10.1149/2754-2726/ae1e10)
Supplement: SI [file NIHMS2127736-supplement-SI.docx]

**Portable Dynamic Laser Speckle Imaging for Rapid Antimicrobial Susceptibility Testing**

**Authors**

Jinkai Yang,^1,2†^ Keren Zhou,^1,2†^ Landon Hernandez,^1,2^ Chen Zhou,^1,2^ Olena Voloshchuk,^3^ Jasna Kovac,^3^ Aida Ebrahimi,^1,2,4,*^ and Zhiwen Liu^1,2,*^

†These authors contributed equally to this work

### **Affiliations**

1. School of Electrical Engineering and Computer Science, The Pennsylvania State University, University Park, Pennsylvania 16802, United States.
2. Materials Research Institute, The Pennsylvania State University, University Park, Pennsylvania 16802, United States.
3. Department of Food Science, The Pennsylvania State University, University Park, Pennsylvania 16802, United States.
4. Biomedical Engineering, The Pennsylvania State University, University Park, Pennsylvania 16802, United States.

**Email address**

Jinkai Yang: [jxy208@psu.edu](mailto:jxy208@psu.edu)

Keren Zhou: [kxz172@psu.edu](mailto:kxz172@psu.edu)

Landon Hernandez: [loh5098@psu.edu](mailto:loh5098@psu.edu)

Chen Zhou: [cjz5147@psu.edu](mailto:cjz5147@psu.edu)

Olena Voloshchuk: [ozv5017@psu.edu](http://ozv5017@psu.edu)

Jasna Kovac: [jzk303@psu.edu](mailto:jzk303@psu.edu)

Aida Ebrahimi: [sue66@psu.edu](mailto:sue66@psu.edu)

Zhiwen Liu: [zzl1@psu.edu](mailto:zzl1@psu.edu)

### *** Corresponding authors**

Zhiwen Liu: [zzl1@psu.edu](mailto:zzl1@psu.edu)

Aida Ebrahimi: [sue66@psu.edu](mailto:sue66@psu.edu)

**System Cost Comparison**

**Table 1.** A comparison of components cost of this work with DLSI.

| pDLSI | **DLSI** |
| --- | --- |
| Laser : DigiKey 1528-1391-ND ~ $5.95 | Laser : THORLABS HNLS008L ~ $1337.5 |
| Camera: iPhone 13 Pro ~ $380 | Camera: Zyla, Andor ~ $7500 |
| Optical components ~ $140 | Optical components ~ $450 |
| subtotal 1 ~ $525.95 | subtotal 2 ~ $9287.5 |

Table 1 summarizes the cost comparison between the pDLSI and conventional DLSI systems.^1^ When considering the light source, imaging sensor, and essential optical components, the total cost of the pDLSI system is approximately 18 times lower, based on the ratio of subtotal 2 to subtotal 1.

**PCA Visualization**

We first recorded a movie (i.e., time series) of dynamical speckles projected onto the paper screen using a smartphone camera. Each movie comprised 240 frames, capturing the dynamic fluctuations in the speckle pattern caused by bacterial motion. the temporal characteristics were analyzed to assess bacterial responses to antibiotic exposure. Fourier transform was performed along the time axis at every pixel. Figure S1 presents the PCA plots (first three principal components) of *E. coli* samples exposed to varying concentrations of gentamicin over 1, 2, and 3 hours. After 1 hour of exposure, no clear separation is observed between the samples. By 2 hours, samples treated with gentamicin concentrations above and below 1× MIC begin to separate and form different clusters. After 3 hours of treatment, the clustering effect becomes pronounced, with separation between bacterial suspension groups exposed to gentamicin concentrations above and below the MIC. These visualizations demonstrate the capability of pDLSI to rapidly distinguish bacterial responses to antibiotics, achieving noticeable differentiation within 2–3 hours.

**Normalization Methods**

The normalization method follows closely our previous work.^2^ Each Fourier spectrum was initially normalized to its DC (0 Hz) component, ensuring consistent scaling across samples (Figure S2a). This DC normalization helped mitigate experimental noise but did not provide clear separation between bacterial groups treated with different antibiotic concentrations, especially when combining data from independent experiments conducted on separate days. To address this, Z-score normalization was applied to the DC-normalized data for each experimental day. Specifically, spectra from all four antibiotic concentration groups (0.25×, 0.5×, 1×, and 2× MIC) were pooled within each day and normalized using the Z-score method for each frequency component. Figure S2b shows the resulting average frequency response plots after Z-score normalization, where bacterial samples exposed to sub-MIC concentrations consistently clustered above the red dotted line, while those exposed to concentrations at or above 1× MIC clustered below it.

**Prediction Results**

Figure S3 illustrates the pixel-level prediction results for bacterial cultures treated with varying antibiotic concentrations across three independent testing sets. Each column corresponds to a specific bacteria-antibiotic pairing: *E. coli* exposed to ampicillin, *E. coli* exposed to gentamicin, and *E. faecalis* exposed to ampicillin, all prepared at an initial concentration of 5×10^5^ CFU/mL. The first row displays results from models trained on data from the second and third experiments and tested on the first experiment. The second row shows predictions using models trained on the first and third experiments, tested on the second experiment. The third row illustrates results from models trained on the first and second experiments and tested on the third experiment. For each bacteria-antibiotic pair, the model predicts the proportions of non-inhibited and inhibited samples at varying antibiotic concentrations: 0.25× MIC, 0.5× MIC, 1× MIC, and 2× MIC. The results demonstrate the model’s capability to accurately distinguish bacterial inhibition levels across different antibiotic concentrations, even when tested on previously unseen experimental datasets. This highlights the robustness of the predictive framework in handling variability across independent experimental conditions.


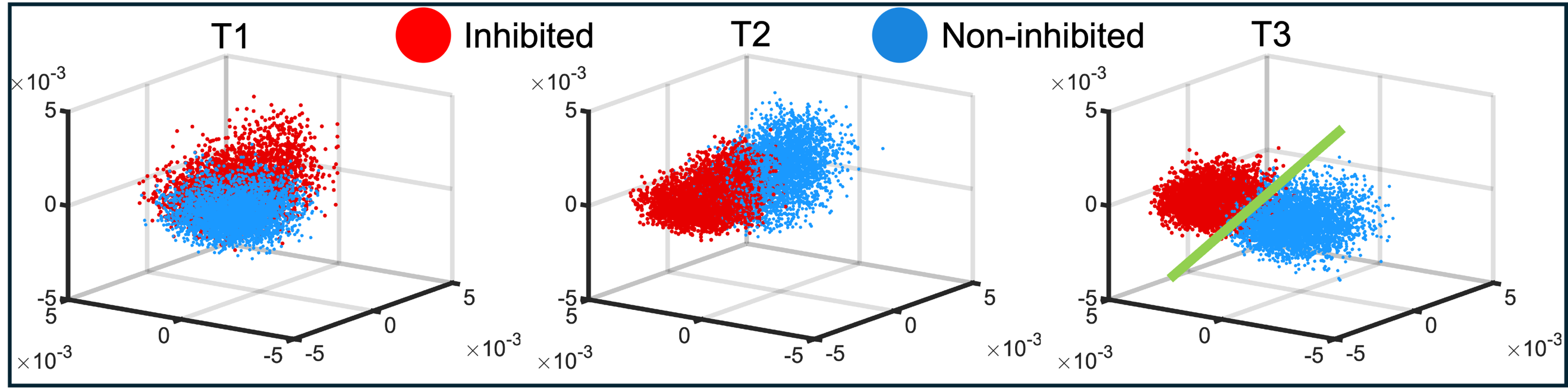


Fig. S1. Principal component analysis (PCA) visualization of E. coli response to gentamicin over time using pDLSI. Bacterial samples were prepared at ~5 × 10⁵ CFU/mL. Red dots represent inhibited populations (≥ MIC), and blue dots represent non-inhibited populations (< MIC). At 1 hour (T1), the two populations are not clearly separable. By 2 hours (T2), partial clustering emerges, indicating early differentiation. At 3 hours (T3), the two populations are distinctly separated, demonstrating that pDLSI enables accurate susceptibility classification within a 2–3-hour window.


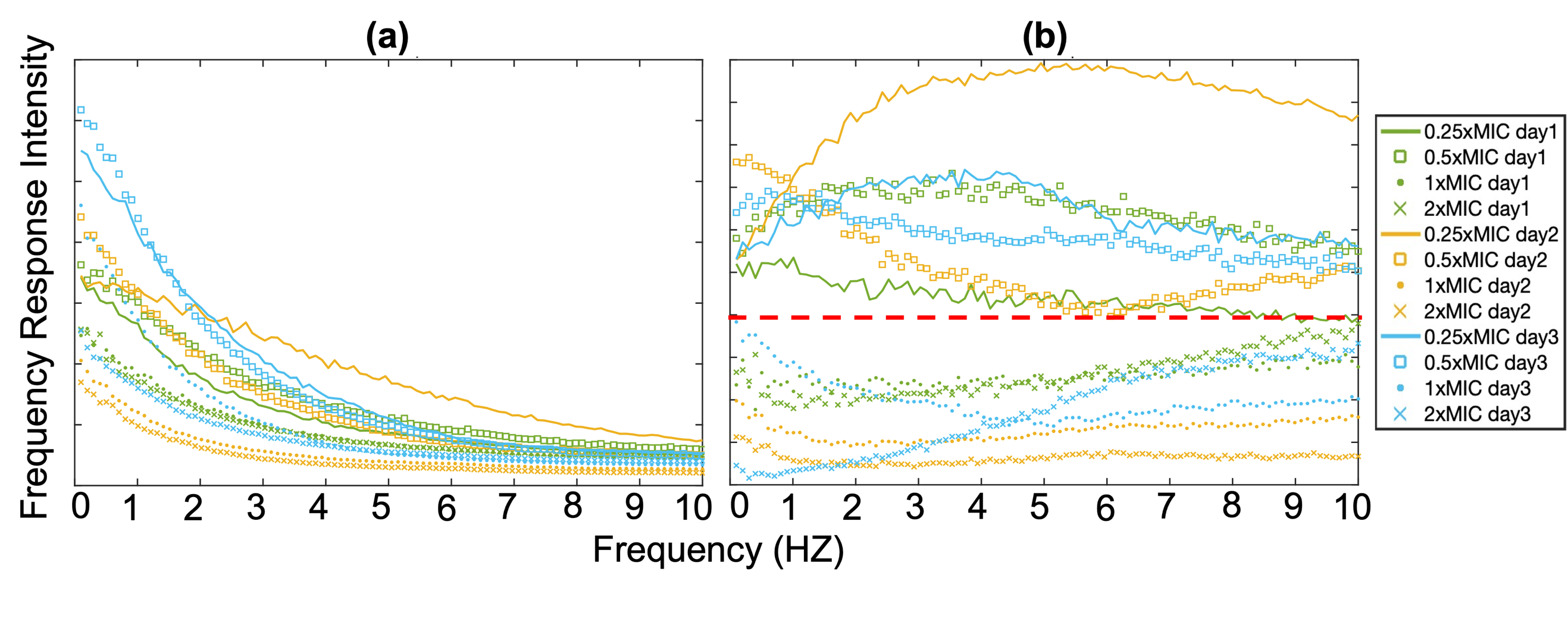


Fig. S2. Average frequency response plots for E. faecalis exposed to ampicillin at an initial concentration of 5×10⁵ CFU/mL for 3 hours. The plots illustrate the frequency response intensities of samples treated with varying antibiotic concentrations: 0.25× MIC (solid line), 0.5× MIC (square line), 1× MIC (solid dot), and 2× MIC (cross sign). Data from independent experiments conducted over three separate days are represented by different colors. **(a)** Frequency response intensities normalized at 0 Hz show overlap between data across all three days, making separation between groups unclear. **(b)** Z-score normalization applied after DC normalization reveals distinct separation, where samples exposed to sub-MIC concentrations are located above the red dotted line, and samples exposed to concentrations at or above 1× MIC fall below the red dotted line.


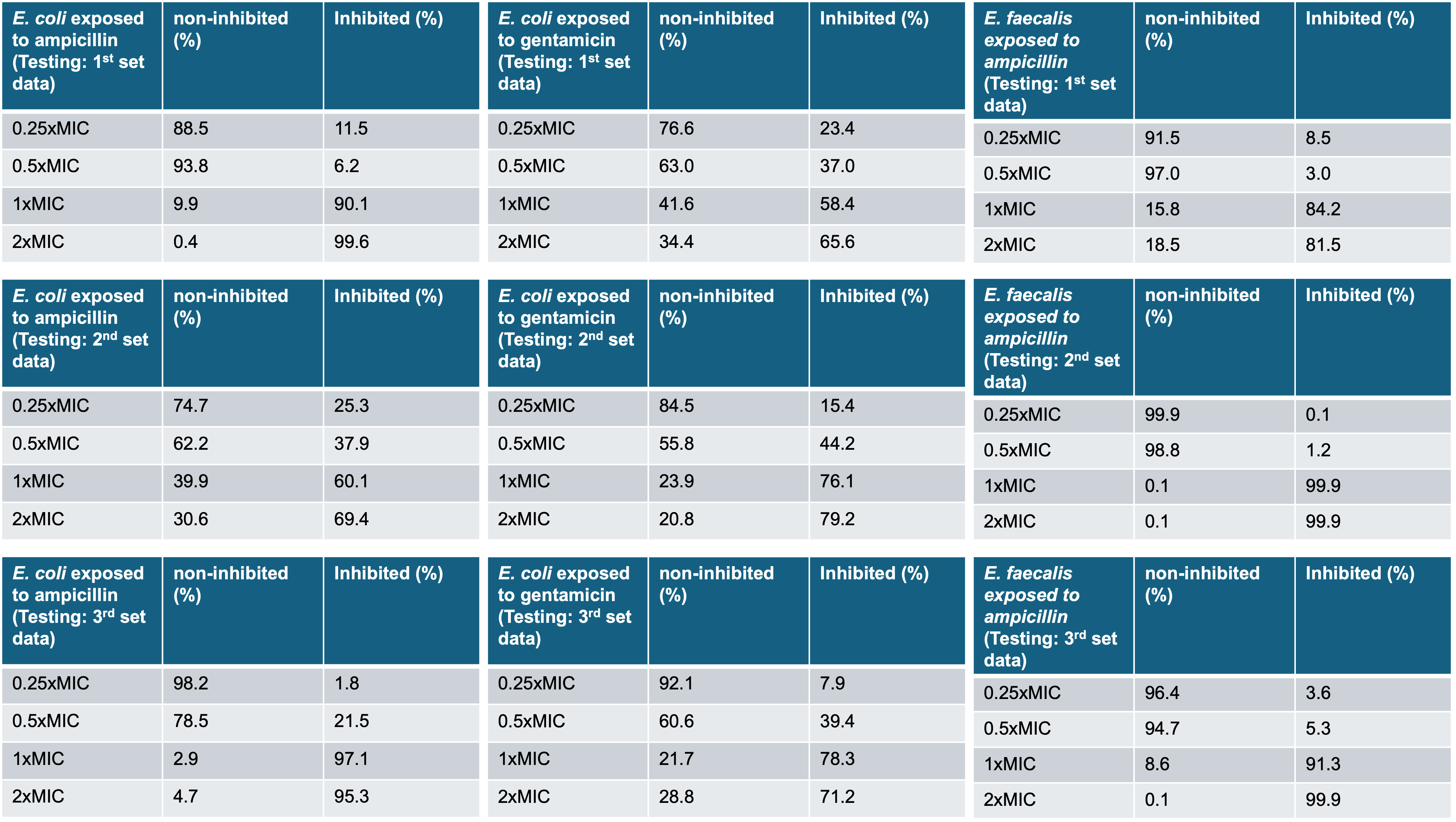


Fig. S3. Inhibition prediction results for bacterial cultures treated with varying concentrations of antibiotics. Each column represents a bacteria-antibiotic pairing: *E. coli* exposed to ampicillin, *E. coli* exposed to gentamicin, and *E. faecalis* exposed to ampicillin, all at an initial concentration of 5×10^5^ CFU/mL. The first row shows prediction results for testing on data from the first experiment, with models trained on data from the second and third experiments. The second row presents prediction results for testing on the second experiment's data, with models trained on the first and third experiments. The third row demonstrates prediction results for testing on data from the third experiment, with models trained on the first and second experiments.

References

(1) Zhou, K.; Zhou, C.; Sapre, A.; Pavlock, J. H.; Weaver, A.; Muralidharan, R.; Noble, J.; Chung, T.; Kovac, J.; Ebrahimi, A.; Liu, Z. Dynamic Laser Speckle Imaging Meets Machine Learning to Enable Rapid Antibacterial Susceptibility Testing (DYRAST). *ACS Sens* **2020**, *5* (10), 3140–3149. https://doi.org/10.1021/acssensors.0c01238.

(2) Yang, J.; Zhou, K.; Zhou, C.; Khamsi, P. S.; Voloshchuk, O.; Hernandez, L.; Kovac, J.; Ebrahimi, A.; Liu, Z. Label-Free Rapid Antimicrobial Susceptibility Testing with Machine-Learning Based Dynamic Holographic Laser Speckle Imaging. *Biosens Bioelectron* **2025**, *278*, 1–9. https://doi.org/10.1016/j.bios.2025.117312.
